# A meta-analysis of the incidence proportion of obesity and associated risk factors in patients with schizophrenia

**DOI:** 10.3389/fpsyt.2026.1818904

**Published:** 2026-04-27

**Authors:** Jianmei Long, Bo Yang, Qiong Zheng

**Affiliations:** Department of Nursing, Chongqing Mental Health Center, Chongqing, China

**Keywords:** incidence proportion, meta-analysis, obesity, risk factors, schizophrenia

## Abstract

**Objective:**

This meta-analysis aimed to investigate the incidence proportion of obesity and its influencing factors in patients with schizophrenia (SCH).

**Methods:**

A comprehensive literature search was conducted using a combination of MeSH subject headings and free-text terms across multiple databases, including PubMed, Web of Science, Embase, Cochrane Library, CNKI, Wanfang Data, VIP Database, and SinoMed. The search covered all publications up to May 26, 2025. Meta-analyses were performed using RevMan 5.4 and Stata 18.0 software.

**Results:**

A total of 18 studies involving 9, 351 patients with SCH were included in the analysis. This meta-analysis revealed that the incidence proportion of obesity in patients with SCH was 33.0%. Additionally, the following factors were significantly associated with an elevated risk of obesity in this population (*P* < 0.05): female sex (*OR* = 1.14, 95% *CI* = 1.10–1.20), elevated FG (*OR* = 1.08, 95% *CI* = 1.04–1.12), diabetes (*OR* = 2.36, 95% *CI* = 1.79–3.10), elevated TG (*OR* = 1.13, 95% *CI* = 1.08–1.18), high LDL (*OR* = 1.88, 95% *CI* = 1.45–2.44), olanzapine use (*OR* = 7.40, 95% *CI* = 4.98–11.00), combined antipsychotic therapy (*OR* = 3.19, 95% *CI* = 2.31–4.41), use of typical antipsychotics (*OR* = 1.46, 95% *CI* = 1.18–1.82), use of atypical antipsychotics (*OR* = 1.70, 95% *CI* = 1.42–2.03), high red blood cell count (*OR* = 2.80, 95% *CI* = 1.60–4.90), and low HDL (*OR* = 1.75, 95% *CI* = 1.41–2.16).

**Conclusion:**

Current evidence indicates that the major risk factors for obesity in patients with SCH include female sex, elevated FG, diabetes, high TG levels, elevated LDL, use of olanzapine, combined antipsychotic treatment, use of typical antipsychotics, use of atypical antipsychotics, higher red blood cell count, and reduced HDL levels. Clinicians should implement early screening and targeted interventions to mitigate the development of obesity in this population, thereby improving their overall quality of life.

**Systematic Review Registration:**

https://www.crd.york.ac.uk/PROSPERO/, identifier CRD420251121298.

## 1 Introduction

An epidemiological survey shows that the lifetime prevalence of schizophrenia (SCH) is approximately 1% (1), SCH is a chronic and persistent disorder characterized by high rates of relapse and disability (2). Most patients require long-term pharmacological treatment, often involving atypical antipsychotic medications (3).Owing to various factors, including psychiatric symptoms and the use of antipsychotic drugs, individuals with SCH are at increased risk of nutritional imbalances and are more susceptible to abnormal body weight changes (4). Studies have indicated that the prevalence of obesity in patients with SCH is three to four times higher than that in the general population (5, 6). In China, the rate of comorbid obesity among individuals with SCH is 20.9% (7), compared to 22.1% in East Asia and the Pacific region, and ranging from 25.5% to 60% in European and American countries (8, 9). The high prevalence of obesity not only diminishes patients’ self-esteem and adversely affects medication adherence and disease prognosis, but also elevates healthcare costs, placing a substantial burden on both families and societ (10). Furthermore, obese patients face an increased risk of metabolic disorders, including hyperlipidemia, hypertension, type 2 diabetes, and cardiovascular and cerebrovascular events, which may ultimately reduce life expectancy (5, 11).

Therefore, there is an urgent need to identify risk factors for obesity in patients with SCH and to implement early prevention and management strategies. Such efforts are essential to reduce the incidence of complications, improve quality of life, shorten hospital stays, and decrease treatment costs. Although several studies have investigated risk factors for obesity in this population, significant heterogeneity and inconsistent findings exist across these studies. For instance, Esan et al. (12) and Li et al. (7) identified female sex as a risk factor for obesity in SCH patients, whereas other studies (11, 13) reported no such association. This study employs a meta-analysis to synthesize existing evidence on the incidence proportion and risk factors of obesity in SCH patients, with the goal of providing evidence-based support for early identification and clinical prevention of obesity. This study is registered with PROSPERO under the registration number CRD420251121298.

## 2 Materials and methods

### 2.1 Literature inclusion and exclusion criteria

#### 2.1.1 Inclusion criteria

① Studies in which the diagnosis of schizophrenia (but not FEP or schizophreniform disorder, in example) is based on DSM or ICD criteria, aged 18 years or older, diagnosed based on clear diagnostic criteria or by a physician; ②Risk factors for obesity in patients with SCH were analyzed using multivariate logistic regression, with complete data reported for the odds ratio (OR) and 95% confidence interval (*CI*); ③ Study types: cohort, case-control, or cross-sectional studies.

#### 2.1.2 Exclusion criteria

① Studies from which original data could not be extracted or converted; ② Reviews, conference abstracts, case reports, or other non‐original research publications; ③ Duplicate publications or studies for which the full text was unavailable; ④ Articles published in languages other than Chinese or English; ⑤ Low‐quality studies.

### 2.2 Literature search strategy

A systematic computer-based search was conducted in the following databases: Web of Science, Cochrane Library, PubMed, Embase, SinoMed, CNKI, VIP Database, and Wanfang Database. The search covered all records from the inception of each database up to May 26, 2025. The English search terms included: overweight/obesity; SCH/Schizophrenia/Schizophrenic Disorder*/Disorder*, Schizophrenic;factor*/influencing factor*/influence factor*/risk factor*/predictor*.The Chinese search terms included: obesity/weight/body mass, schizophrenia/schizophrenic disorder, risk factor/influencing factor/risk factor/predictive factor. The search strategy combined both subject headings and free-text terms, and employed a reference-tracking approach to identify additional relevant publications.

### 2.3 Literature screening and data extraction

Two researchers independently performed literature screening and data extraction, followed by cross-checking. Any disagreements were resolved through discussion with a third researcher. The screening process involved the following steps: all retrieved records were initially reviewed to remove duplicates; titles and abstracts were then screened for eligibility; finally, the full texts of potentially relevant studies were assessed for final inclusion. The investigators reviewed relevant meta-analytic literature and developed a dedicated data extraction form in Microsoft Excel 2021. Data from the finally included studies were extracted independently using this form, and the extracted items included: first author, year of publication, study location, study design, sample size, number of obesity cases, incidence proportion, age of participants, reported risk factors, and effect size (OR and 95% CI).

### 2.4 Quality assessment of included studies

Two researchers independently assessed the methodological quality of the included studies using appropriate bias risk assessment tools. Any discrepancies in evaluation were resolved through discussion. The Newcastle-Ottawa Scale (NOS) was used to evaluate the quality of case-control and cohort studies, with a maximum score of 9. Studies scoring ≥7, 4–6, and <4 were considered high, moderate, and low quality, respectively. For cross-sectional studies, the assessment was performed using the criteria recommended by the US Agency for Healthcare Research and Quality (AHRQ), which comprises 11 items and a maximum score of 11. Scores of ≥8, 4–7, and <4 indicated high, moderate, and low quality, respectively. Only studies rated as moderate or high quality were included in this meta-analysis.

### 2.5 Statistical analysis

Meta-analyses were performed using Stata 17.0 (for incidence proportion of obesity) and RevMan 5.4 (for risk factors). We conducted meta-analyses for risk factors reported in at least 2 studies with consistent outcome definitions. The strength of association was estimated using the OR and 95% *CI*. Heterogeneity among studies was assessed using the I² statistic. A fixed-effects model was applied when *P* > 0.1 and I² ≤ 50%; otherwise, a random-effects model was used. Potential sources of heterogeneity were explored through sensitivity analyses using the exclusion method. For analyses involving three or more studies, publication bias was evaluated using Egger’s test in Stata 17.0. A P-value > 0.05 was considered indicative of no significant publication bias. Factors reported in only a single study or with incompatible data types were summarized descriptively. Subgroup analyses were conducted based on the characteristics of the included studies to examine potential sources of heterogeneity in obesity incidence proportion. Sensitivity analysis was performed using the leave-one-out method. A significance level of α = 0.05 was adopted for all statistical tests.

## 3 Results

### 3.1 Literature search results

**Figure 1:**
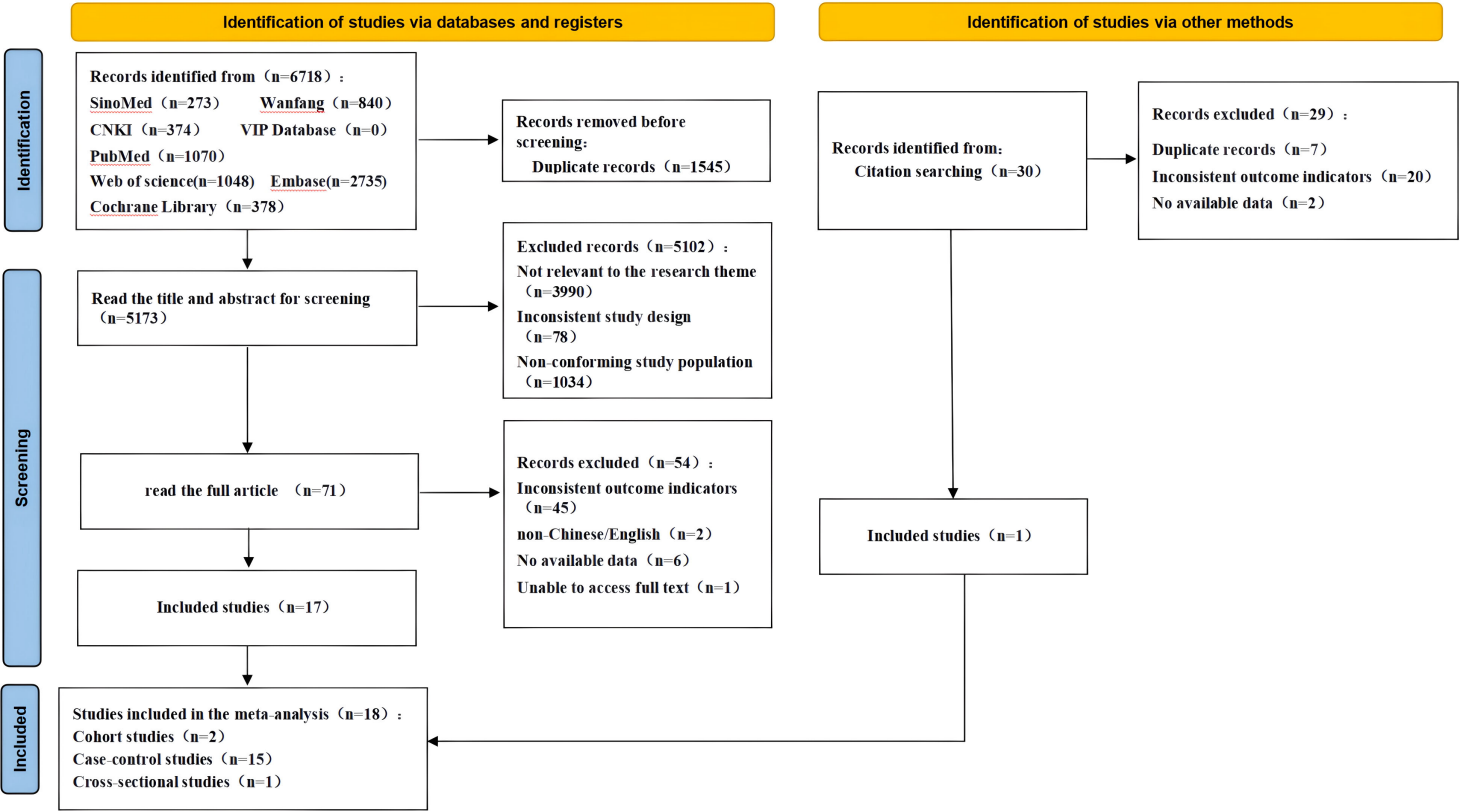
Flowchart of study selection.

A total of 6,718 articles were initially retrieved. After removing duplicates using EndNote software, 5,173 articles remained. Following a preliminary screening based on titles and abstracts, 71 articles were retained for full-text review. After evaluating the full texts, 45 for inconsistent outcome indicators, 6 due to incomplete data, 1 due to unavailability of the full text, and 2 for being in a language other than Chinese or English. Ultimately, 18 articles met the inclusion criteria (4, 7, 11, 13–24), including 10 in Chinese (4, 14–21, 25) and 8 in English (7, 11–13, 22–24, 26). The literature screening process is summarized in **Figure 1**.

### 3.2 Basic characteristics and quality assessment of included studies

The 18 included studies comprised 3 cohort studies (4, 20, 26), 1 case-control study (16), and 14 cross-sectional studies (7, 11–15, 17–19, 21–25). All studies were rated as moderate to high quality based on the respective quality assessment tools. The basic characteristics and methodological quality evaluation results are summarized in **Table 1**.

**Table 1:** presents the basic characteristics and methodological quality assessment results of the included studies (n = 18).

First author	Country	Year	Total/n	Cases/n	Incidence proportion/%	Age	Follow-up time	Risk factors	NOS/ AHRQ score
Li, Y (21)	China	2023^1)^	278	127	45.7	≥18	—	⑮⑯⑰⑱⑲⑳	5
Lin, F (20)	China	2022^3)^	247	38	15.4	46.89 ± 9.692	—	②④⑦⑧	7
Liu, F (19)	China	2020^1)^	210	129	61.43	18-60	—	㉑㉒	6
Liu, J (18)	China	2018^1)^	180	53	29.4	(51.4 ± 14.1)	—	①②⑤㉓	6
Wang, F (16)	China	2023^2)^	108	57	52.78	52.82 ± 7.44	—	①⑤⑦㉕㉖	7
Yang, X (14)	China	2018^1)^	238	120	50.42	50.72 ± 11.78	—	①㉕㉛㉜	5
Zhang, L (25)	China	2022^1)^	300	115	38.33	51.91 ± 1.92	—	①⑬⑭⑮㉕	6
Wang, J (15)	China	2020^1)^	325	53	16.30	18-75	—	①⑤⑦⑨	7
Bodén, R (26)	Sweden	2009^3)^	59	22	37	18-59	5 years	㉞㉟㊱㊲㊳	9
Kim, M (23)	Korea	2023^3)^	270	53	19.60	40.4 ± 11.1	3 years	㉔㉙	6
Li, Q (7)	China	2017^1)^	206	43	20.90	51.7 ± 7.8	—	①③⑤	7
Esan, O (12)	Nigeria	2021^1)^	207	26	12.60	44.2 ± 8.0	—	①④㉝	7
Huang, X (13)	China	2023^1)^	985	191	19.40	47.2 ± 12.5	—	③⑪	7
Tian, Y (11)	China	2015^1)^	633	104	16.40	47.00 ± 9.74	—	⑤⑨	6
Yong, N (24)	China	2022^1)^	284	161	56.70	51.43 ± 7.91	—	①②③⑤⑦⑨㉗㉚	6
Subramaniam, M (22)	Singapore	2014^1)^	973	716	73.60	41.7 ± 11.0	—	①⑥㉕㉘㊴	8
Liu, Y (17)	China	2023^1)^	3200	550	17.19	58.38(47.50,67.75)	—	②④⑤⑩⑫㉓㉘	5
Shi, Y (4)	China	2025^3)^	401	78	19.50	45(39,52.25)	—	④⑤⑦㉕㉟	8

^1)^Cross-sectional study, 2)Case-control study, 3)Cohort study; ① Female sex、② Fasting glucose (FG)、③ Diabetes、④ Age、⑤ Triglyceride(TG)、⑥ Years of education、⑦ High-density lipoprotein(HDL)、⑧ Negative symptoms、 ⑨ Low-density lipoprotein (LDL)、⑩ Hypertension、⑪ Total Score of Positive and Negative Syndrome Scale(sumPANSP)、 ⑫ Elevated alanine aminotransferase (ALT)、⑬ Clozapine use、 ⑭ Quetiapine use、⑮ Olanzapine use、⑯ Sedentary behavior、⑰ Exercise index/Exercise duration、⑱ Frequent alcohol consumption (within past 3 months)、⑲ Frequent snacking、 ⑳ Frequent smoking (within past 3 months)、 ㉑ Hyperinsulinemia、 ㉒ Hip circumference、 ㉓ Use of atypical antipsychoti、 ㉔ Regular dietary habits、 ㉕ Combined antipsychotic therapy、 ㉖ Longer duration of illness、 ㉗ Waist circumference ≥ 90 cm、 ㉘ Use of typical antipsychoti、 ㉙ Healthy dietary habits、 ㉚ Apolipoprotein B ≥ 0.70 g/L、 ㉛ Family history of obesity、 ㉜ Daily sleep duration、 ㉝ Education level、 ㉞ Hemoglobin level、 ㉟ Red blood cell count、 ㊱ Hematocrit、 ㊲ Elevated γ-glutamyl transferase、 ㊳ Elevated creatinine level、 ㊴ Comorbidities.

### 3.3 Meta-analysis of obesity incidence proportion in patients with SCH

**Figure 2:**
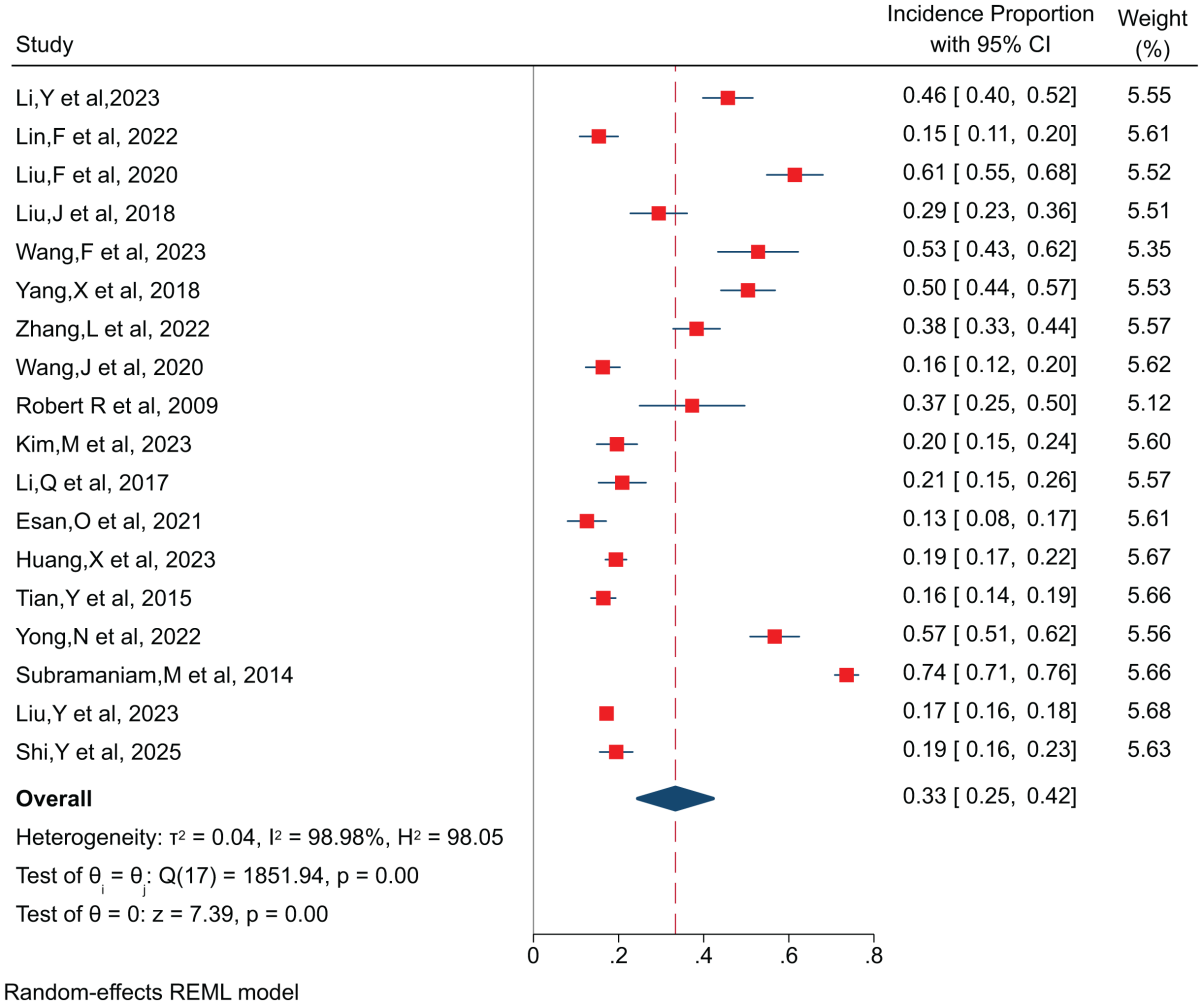
Forest plot of the obesity incidence proportion in patients with SCH.

The meta-analysis of the 18 included studies indicated that the incidence proportion of obesity among patients with SCH ranged from 12.6%~73.6%, with significant heterogeneity observed across studies (*I*² = 97.77%, *P* < 0.0001). A random-effects model was therefore applied to pool the effect estimates. The combined obesity incidence proportion was 33.0% (95*% CI*: 25.0% – 42.0%, *P* < 0.0001), as shown in **Figure 2**.

Subgroup analyses were performed according to region, study type and follow-up time. The results indicated that the incidence proportion was 34.5% in Asia, 12.6% in Nigeria, and 37.3% in Sweden; 35.2% in cross-sectional studies, 52.8% in case-control studies and 20.4% in cohort studies; and the incidence proportion was 19.6% at 3 years and 37.3% at 5 years.

### 3.4 Meta-analysis of risk factors for obesity in patients with SCH

A total of 39 risk factors were examined across the included studies. Among these, 12 factors were reported in two or more studies and were included in the meta-analysis. The remaining 27 factors, each reported in only a single study, were summarized using descriptive analysis. Results of the meta-analysis are presented in **Table 2**.

**Table 2:** Meta-analysis of risk factors for obesity.

Factors	The number of studies included	Heterogeneity test results		Effect model	Meta-analysis results		
		*I*^2^%)	*P*		OR(95%*CI*)	*Z*	*P*
female sex	9	88%	<0.00001	a random-effects model	1.14(1.10-1.20)	5.98	<0.00001
elevated FG	4	88%	<0.00001	a random-effects model	1.08(1.04-1.12)	4.1	<0.00001
diabetes	3	1%	0.36	a fixed-effects model	2.36(1.79-3.10)	6.15	<0.00001
age	4	90%%	<0.0001	a random-effects model	0.99(0.96-1.02)	0.81	0.42
elevated TG	8	90%%	<0.00001	a random-effects model	1.13(1.08-1.18)	5.58	<0.00001
high LDL	3	64%	0.06	a random-effects model	1.88(1.45-2.44)	4.73	<0.00001
olanzapine use	2	89%	0.002	a random-effects model	7.40(4.98-11.00)	9.90	<0.00001
combined antipsychotic therapy	5	82%	<0.0002	a random-effects model	3.19(2.31-4.41)	7.03	<0.00001
use of typical antipsychotics	2	0%	0.49	a fixed-effects model	1.46(1.18-1.82)	3.42	0.0006
use of atypical antipsychotics	2	65%	0.09	a random-effects model	1.70(1.42-2.03)	5.83	<0.00001
high red blood cell count	2	0%	0.79	a fixed-effects model	2.80(1.60-4.90)	3.60	0.0003
low HDL	5	88%	<0.00001	a random-effects model	1.75(1.41-2.16)	5.13	<0.00001

#### 3.4.1 Laboratory examination factors

Four studies (17, 18, 20, 24) examined the association between elevated FG and obesity. Significant heterogeneity was observed (I² = 88.00%, *P* < 0.0001), and a random-effects model was applied. The pooled result showed an OR of 1.08 (95% CI: 1.04–1.12; *P* < 0.00001). Eight studies (4, 7, 11, 15–18, 24) reported on the relationship between elevated TG levels and obesity. Due to high heterogeneity (I² = 90%, *P* < 0.0001), a random-effects model was used, yielding an OR of 1.13 (95% CI: 1.08–1.18; *P* < 0.00001). Three studies (11, 15, 24) investigated the association between LDL and obesity. Moderate heterogeneity was present (I² = 64%, *P* = 0.06), and a random-effects model produced an OR of 1.88 (95% CI: 1.45–2.44; *P* < 0.001). Two studies (4, 26) evaluated the effect of red blood cell count on obesity. With low heterogeneity (I² = 0%, *P* = 0.79), a fixed-effects model was used, resulting in an OR of 2.80 (95% CI: 1.60–4.90; *P* < 0.001). Five studies (4, 15, 16, 20, 24) assessed the link between HDL and obesity. Significant heterogeneity was detected (I² = 88%, *P* < 0.00001), and a random-effects model gave an OR of 1.75 (95% CI: 1.41–2.16; *P* < 0.00001).

#### 3.4.2 Treatment factors: pharmacotherapy

Two studies (21, 25) examined the association between olanzapine use and obesity. Significant heterogeneity was observed (I² = 89.00%, *P* = 0.002), and a random-effects model was applied. The pooled analysis yielded an OR of 7.40 (95% CI: 4.98–11.00; *P* < 0.00001). Five studies (4, 14, 16, 22, 25) investigated the relationship between combined antipsychotic therapy and obesity. Due to substantial heterogeneity (I² = 82.00%, *P* < 0.0002), a random-effects model was used, resulting in an OR of 3.19 (95% CI: 2.31–4.41; *P* < 0.00001). Two studies (17, 22) evaluated the effect of typical antipsychotic use on obesity. With low heterogeneity (I² = 0%, *P* = 0.49), a fixed-effects model was employed, showing an OR of 1.46 (95% CI: 1.18–1.82; *P* < 0.001). Two studies (17, 18) assessed the association between atypical antipsychotic use and obesity. Moderate heterogeneity was present (I² = 65.00%, *P* = 0.09), and a random-effects model produced an OR of 1.70 (95% CI: 1.42–2.03; *P* < 0.00001).

#### 3.4.3 Other factors

Nine studies (7, 12, 14–16, 18, 22, 24, 25) examined the influence of sex on obesity in patients with SCH. Significant heterogeneity was observed (I² = 88%, *P* < 0.00001). A random-effects model analysis indicated that female sex was a risk factor for obesity, with an OR of 1.14 (95% CI: 1.10–1.20; *P* < 0.00001). Three studies (7, 13, 24) investigated the association between diabetes mellitus and obesity. With low heterogeneity (I² = 1%, *P* = 0.36), a fixed-effects model was applied, yielding an OR of 2.36 (95% CI: 1.79–3.10; *P* < 0.00001). Additional factors reported in single studies as potentially associated with obesity in SCH patients include: years of education, negative symptoms, hypertension, total PANSS score, elevated ALT, use of clozapine, use of quetiapine, sedentary behavior, exercise index/duration, frequent alcohol consumption (within the past 3 months), frequent snacking, frequent smoking (within the past 3 months), hyperinsulinemia, hip circumference, regular dietary habits, longer disease duration, waist circumference ≥ 90 cm, healthy dietary habits, apolipoprotein B ≥ 0.70 g/L, family history of obesity, daily sleep duration, education level, hemoglobin level, hematocrit, γ-glutamyl transferase, elevated creatinine level, and presence of comorbidities. As each of these factors was reported in only one study, meta-analysis was not feasible.

### 3.5 Sensitivity analysis

Sensitivity analysis, conducted by sequentially excluding each study, demonstrated that the overall incidence proportion remained stable, supporting the robustness of the findings. Sensitivity analysis was performed using the leave-one-out method to assess the impact of individual studies on heterogeneity. The results showed that the heterogeneity of low-density lipoprotein and high-density lipoprotein decreased after the removal of reference (24). In contrast, the heterogeneity associated with female sex, elevated FG, diabetes, elevated TG, and combined antipsychotic treatment showed no significant changes, suggesting that the results for these factors are stable and highly reliable. For factors reported in only two studies-namely, olanzapine use, typical antipsychotic use, atypical antipsychotic use, and red blood cell count-sensitivity analysis using the leave-one-out method was not feasible due to the limited number of available studies.

### 3.6 Detection of publication bias

When the number of studies included for the incidence variable was greater than 10, the scatter points in the funnel plot showed noticeable asymmetry. However, Egger’ s test indicated no statistically significant difference (*t* = 1.52, *P* = 0.149), suggesting a low likelihood of publication bias. For individual risk factors, the number of included studies was fewer than 10, so no publication bias analysis was conducted.

## 4 Discussion

### 4.1 The incidence proportion of obesity is higher in patients with SCH

A meta-analysis of 18 studies reporting the incidence proportion of obesity in patients with SCH yielded a pooled incidence proportion of 33.0% (95% CI: 25.0%–42.0%), which is consistent with the 37% prevalence reported by Bodén et al (26). However, this meta-analysis exhibited significant heterogeneity, which may be attributed to variations in sample sizes across the included studies. Additionally, most of the studies included in this analysis did not report the duration of follow-up, precluding the calculation of true incidence rates. Consequently, our estimates represent incidence proportions, which may overestimate the risk compared with time-adjusted incidence rates.

Subgroup analysis indicated regional differences in the incidence proportion of obesity among SCH patients, This discrepancy may be associated with the number of included studies, variations in healthcare standards across regions, and dietary or lifestyle habits; The incidence proportion of obesity in patients increased progressively with the extension of follow-up duration, the incidence proportion was 19.6% at 3 years and 37.3% at 5 years. This phenomenon may be ascribed to the combined effects of persistent exposure to antipsychotic agents, disease-associated negative symptoms, and an underlying metabolic disturbance during long-term follow-up, all of which contribute to the progressive elevation of obesity incidence proportion with disease progression. This phenomenon may be ascribed to the combined effects of persistent exposure to antipsychotic agents, disease-associated negative symptoms, and an underlying metabolic disturbance during long-term follow-up, all of which contribute to the progressive elevation of obesity incidence proportion with disease progression.

The higher prevalence of obesity in SCH patients may be attributed to various risk factors such as gender, concomitant use of antipsychotic medications, and elevated TG levels (4, 7). Another study (27) indicated that prolonged illness can significantly impact patients’ eating habits, often manifesting as loss of appetite with difficulty eating, intermittent episodes of binge eating, coupled with reduced inclination for physical activity and significantly decreased exercise levels, all of which may contribute to substantial weight gain. Furthermore, the higher obesity prevalence among SCH patients might also be related to insufficient attention from healthcare providers toward obesity in this population. Therefore, mental health professionals should prioritize SCH patients by enhancing early screening and prevention of obesity risks.

### 4.2 SCH patients have multiple risk factors for obesity, which necessitates enhanced identification

This meta-analysis revealed that female sex, elevated red blood cell count, and diabetes are risk factors for obesity in patients with SCH. The higher prevalence of obesity among female SCH patients may be attributed to disruptions in estrogen metabolism. Both the onset of SCH and the use of antipsychotic medications can contribute to decreased estrogen levels in the body, which reduces fat breakdown and utilization, leading to increased fat accumulation particularly in the abdominal area (16). Impaired glucose tolerance is present in patients with SCH. In the general population, hemoglobin and hematocrit have been proposed as surrogate markers of insulin resistance, which can predict the development of obesity (26). Therefore, gender-specific intervention strategies should be developed, with an emphasis on blood biochemical monitoring and diabetes management, to prevent the onset of obesity.

This study found that higher FG, TG, and LDL levels, as well as lower HDL levels, are risk factors for obesity in patients with SCH. Li et al. (28) indicated that metabolic dyslipidemia may be closely associated with the use of antipsychotic medications. They also observed that patients on long-term second-generation antipsychotics had higher blood glucose levels compared to those taking first-generation antipsychotics. This leads to significantly elevated fasting blood glucose and fasting insulin levels in SCH patients. Hyperinsulinemia is a high-risk factor for obesity, as insulin promotes fat synthesis and storage, reduces free fatty acids in the blood, and inhibits fat breakdown and oxidation (29, 30). Additionally, Mizuki et al. (31, 32) found that SCH patients often exhibit insulin resistance. Due to this resistance, glucose uptake and utilization are reduced, leading to the conversion of excess glycogen into fat, which subsequently contributes to weight gain. Therefore, early detection of abnormal glucose and lipid metabolism indicators and the implementation of effective interventions can help prevent the development of obesity.

The use of atypical antipsychotics, typical antipsychotics, olanzapine specifically, and combination therapy with antipsychotic drugs are risk factors for obesity in SCH patients. Patients treated with atypical antipsychotics exhibit higher levels of blood glucose, low-density lipoprotein cholesterol, and TG compared to those receiving typical antipsychotics, thereby increasing the risk of obesity in SCH patients (7). This difference may be attributed to the fact that patients on typical antipsychotics receive significantly higher daily doses (in chlorpromazine equivalents) than those on atypical agents (11). Among the various risk factors, olanzapine exerted the most pronounced effect on weight gain in patients with schizophrenia, which is consistent with the findings of Huhn M et al. (33). Our meta-analysis revealed that the risk with olanzapine monotherapy was (OR = 7.40, 95% CI: 4.98–11.00), compared with (OR = 3.19, 95% CI: 2.31–4.41) for combined antipsychotic therapy. This finding indicates that patients with schizophrenia receiving olanzapine monotherapy are at a significantly higher risk of obesity than those on antipsychotic polypharmacy regimens. In the setting of antipsychotic polypharmacy, the dosage of olanzapine may be appropriately reduced, thereby mitigating the metabolic adverse effects associated with its monotherapy. This observation underscores the potential advantage of antipsychotic polypharmacy in reducing metabolic side effects and provides a valuable reference for clinical decision-making. Among various antipsychotics, olanzapine has the most pronounced effect on body weight in SCH patients. Antipsychotic-induced weight gain is associated with transient elevations in serum transaminases (26). Furthermore, weight gain is significantly correlated with the reduction of psychopathological symptoms during antipsychotic treatment. A possible explanation is that antipsychotics influence weight gain through serotonin neurotransmission, as serotonergic signaling pathways may play a role in appetite regulation. and 5HT2C receptor blocking is directly related to the weight gain induced by antipsychotic drugs and improvement of clinical symptoms (34).The impact and safety of antipsychotic medications on obesity require further investigation in future studies.

This study indicates that age is not a risk factor for obesity in patients with SCH. This finding may be attributed to the limited number of included relevant studies, which could have led to discrepancies in the results. Further exploration is needed to clarify the relationship between age and obesity in the future. Although other antipsychotics (e.g., clozapine, risperidone, quetiapine) are also associated with an increased risk of obesity (35), only olanzapine was found to exert a significant effect in the final meta-analysis. This finding was mainly attributable to two factors: first, only one study was available for both clozapine and quetiapine separately; second, extractable estimates of obesity risk (e.g., OR, HR) for risperidone were lacking across all included studies, which precluded its inclusion in the meta-analysis. Previous studies have indicated that the metabolic adverse effects of olanzapine are more pronounced in the short term, whereas the obesity risk associated with agents such as risperidone requires a longer follow-up period for observation (36, 37). However, only two studies included in our meta-analysis reported follow-up data, and thus the obesity risk of other antipsychotics could not be identified in the present analysis. The obesity risk of other antipsychotics therefore warrants equal attention.

## 5 Limitations

This meta-analysis has several limitations: ① The study only retrieved literature from Chinese and English databases, and the number of included studies for some risk factors was relatively small, which may affect the comprehensiveness of the findings. The results of the meta-analysis still require further validation. ② The sample sizes of the included studies varied significantly, ranging from 59 to 3,200 cases, this may represent a key source of heterogeneity across the included studies, which could to some extent compromise the stability and precision of the pooled results. A random-effects model was therefore applied for data pooling to mitigate such heterogeneity. ③ Furthermore, none of the included studies reported the mean or median follow-up duration. Meanwhile, subgroup analyses for the 3-year and 5-year follow-up were each based on only one study, as the remaining studies could not be included in the analysis due to the absence of relevant follow-up duration data. This constitutes a major limitation of the present study. It is recommended that standardized reporting of follow-up duration be adopted in future research.

## 6 Conclusion

This meta-analysis identified that the following factors are associated with a higher incidence of obesity in patients with SCH: female sex, elevated FG, diabetes, increased TG levels, higher LDL, use of olanzapine, combined antipsychotic therapy, use of typical antipsychotics, use of atypical antipsychotics, elevated red blood cell count, and lower HDL levels. Healthcare providers should dynamically assess the risk of obesity in SCH patients and implement proactive nursing measures targeting modifiable factors to reduce the incidence of obesity as much as possible and improve patients’ quality of life. Clinical practitioners may refer to the findings of this study to develop obesity risk assessment tools tailored to the characteristics of SCH patients and construct obesity risk prediction models, thereby providing evidence-based support for targeted interventions.

## Data Availability

The original contributions presented in the study are included in the article/**Supplementary Material**. Further inquiries can be directed to the corresponding authors.
